# Perspectives: involving persons with lived experience of mental health conditions in service delivery, development and leadership

**DOI:** 10.1192/bjb.2021.51

**Published:** 2022-06

**Authors:** Charlene Sunkel, Claudia Sartor

**Affiliations:** Global Mental Health Peer Network, Roodepoort, South Africa

**Keywords:** Mental health conditions, World Health Organization, service delivery, developmental disorders, leadership

## Abstract

Globally, there has been an emphasis on the importance and value of involving people with lived experience of mental health conditions in service delivery, development and leadership. Such individuals have taken on various roles, from peer support specialists and other specialised professions to leadership in mainstream industries. There are, however, still obstacles to overcome before it is possible to fully include people with lived experience at all levels in the mental health and related sectors. This article discusses the benefits, both to the individual and to the public, of involving persons with lived experience in service delivery, development and leadership.

The World Health Organization (WHO) describes advocacy as ‘an important means of raising awareness on mental health issues and ensuring that mental health is on the national agenda of governments. Advocacy can lead to improvements in policy, legislation and service development’.^1^ By this definition, people with lived experience of mental health conditions (hereafter referred to as ‘persons with lived experience’) have increasingly become the driving force behind advocacy and awareness work globally, and have entered the mental health workforce in various capacities of service delivery, from peer-to-peer support work to professional occupations in the field of mental health and human rights.

The all-encompassing objective of lived experience involvement is development and leadership and to create communities in which people with lived experience are able not only to survive but rather thrive with a mental health condition.

## Obstacles for lived experience involvement in service delivery, development and leadership

Despite the growing acknowledgement of the value of lived experience, there are still a number of obstacles to overcome before it is possible to fully include people with lived experience at all levels.

- We are embedded in frameworks of shared values and beliefs – social, cultural and environmental factors that directly and indirectly influence psychopathology.^2^ Our experiences throughout our life journeys largely determine the nature of our perceptions and our attitudes towards notions. Although there is undoubtedly a complex connection between culture and mental illness, negative perceptions and insensitivity result in stigma, discrimination and violations of human rights.
- The language used to talk about mental health also creates barriers because the way in which we understand, and subsequently express, experiences can be easily misinterpreted and consequently negative assumptions are made about others. It is therefore imperative to obtain clarity in the distinction between terms such as ‘mental health’ and ‘mental illness’.^3^
- People continue to believe that individuals with mental health conditions are violent, mad, lazy, possessed by demons^4^ and incapable of positively contributing to society and the economy. Negative stereotypes and inaccurate portrayals such as these affect help-seeking behaviour and result in an unwillingness of those suffering to seek support from loved ones. People ought to be cognisant that when they perpetrate and share negative stereotypes, it has an impact on the entire recovery process and it affects not only those who struggle with mental illness but also those working in the mental health field. The fact is that negative perceptions lead to prejudice at home, in the workplace, within local communities and within our healthcare systems.^3^

Apart from these attitudinal barriers, social determinants of mental illness,^5^ including poor education, unemployment and poverty, further hinder opportunities for people with lived experience to develop leadership potential, pursue their passion for mental health work and follow a career in mental health advocacy and service delivery. Specifically, poor education along with a lack of access to adequate information are contributing factors that result in low mental health and human rights literacy among the more disadvantaged people with lived experience who are subjected to social and economic inequalities.^5^

## Lived experience in development and leadership

Lived experience advocates who have pursued a passion to improve mental healthcare and services have been using their own experiences and recovery journeys as a means to highlight the gaps, not only within the mental healthcare system but also within other societal systems outside of the healthcare sector. Lived experience advocates have, of course, also challenged the status quo and taken a stand on a human rights perspective as the foundation of change, transformation and systems strengthening. Building on this, there is growing recognition of the value of persons with lived experience as agents of change through contributing their unique expertise, in-depth knowledge of navigating systems, first-hand experience of segregation and discrimination, and their ability to provide high-level and practical solutions. This recognition places persons with lived experience as experts by experience and leaders of development and change, through their influence on policy and practice.

At a global level, there is evidence that mental health guidelines, strategies and policies have notably become more consistent in including lived experience perspectives and recognising those experiences as integral to service development, implementation, monitoring and evaluation. Engagement with persons with lived experience is critical to identify the flaws in the system – what others may perceive as a complex breakdown in the system may often be repaired with simple, practical, cost-effective and innovative solutions proposed by the users of that particular system or service.

The Global Mental Health Peer Network (GMHPN), an international lived experience organisation, was established to enhance the voices of persons with lived experience across the world and has a specific focus on leadership development. That focus derived from the recognition of the obstacles (described above) that ultimately deny countries the expertise and value that people with lived experience are able to contribute to improving the accessibility and quality of mental healthcare and services at the local level. The GMHPN's executive structure is designed to develop new global lived experience leadership and create a sophisticated platform for diverse lived experience voices to be at the forefront of change. Empowerment as a critical element in leadership development is built into the GMHPN structure. Lived experience leaders are appointed to the GMHPN Executive Committee as representatives of their respective country or region, and ultimately serve in an advisory capacity, contribute to the development of strategies to achieve common advocacy goals and ultimately influence policy and practice.

The benefits of people with lived experience in leadership positions is evident from the following statements (all statements in this article appear with the permission of those named).

‘Since I have been appointed as the Deputy Representative for the African Region on the GMHPN Regional Executive Committee, it has given me the courage to think big. It has made me a person with a mission, to bring some meaningful changes on how people perceive mental health, not only in my country Zimbabwe but in Africa. This has given me a chance to dream big about how people with psychosocial disabilities throughout the world should live – with dignity and stigma-free.’ (Angelica Mkorongo, Zimbabwe)

‘Learning from shared experiences from other GMHPN members has largely widened my horizon to see what can be achieved through lived experience advocacy. Gatherings of such courageous individuals with high calibre from diverse geographical and cultural settings for the common cause of lived experience advocacy is really eye-opening. This has greatly impacted my understanding of mental health being a global issue. I can definitely certify that my engagement as Country Executive Committee representative for Ethiopia is the greatest empowering, uplifting, and inspiring experience I ever had in my journey as global mental health advocate and as a person with lived experience.’ (Eleni Misganaw, Ethiopia)

‘My position as regional representative for Africa on the GMHPN Regional Executive Committee means a lot for me as a person, and for persons living with mental health conditionsin my country. It is validating of our experiences and an opportunity for me to join other global voices to make mental health matter. It is also a wonderful global opportunity to de-stigmatize mental illness and advocate for better inclusion policies with regard to persons with lived experiences, especially in a region like mine where mental illness is generally considered taboo, due to witchcraft or spiritual attacks.’ (Marie Abanga, Cameroon)

‘My role in the GMHPN on the Regional Executive Committee enables me to empower individuals with lived experiences, bring forth issues such as local laws and civil society support, and bring together the larger issues of universal rights concerning mental health, such as basic income and housing, employment rights and peer and ally support networks. This position allows me to gain insight and mobilize resources to address challenges unique to the cultural understanding of mental health in South East Asia, allowing a culturally relevant solution-focussed approach.’ (Anjali Singla, India)

‘My role on the GMHPN Regional Executive Committee is a vitalizing booster to what sometimes feel like a rocky up-cliff journey, and an active propeller to encourage lived experiences to be part of the strategy in building mentally healthy workplaces. It is crucial to share best practices within and across regions so we do it together, and in a way that respects local cultures and thoughts.’ (Enoch Li, China)

## Lived experience in service delivery

Persons with lived experience have not only been users of services but many have become service providers themselves – something that would never have been imagined just a few decades ago. However, in our experience, even today it is still unimaginable in some countries that someone with a mental health condition can in fact be meaningfully employed, let alone employed within the mental healthcare workforce.

Several GMHPN Executive Committee members (with lived experience) from across the world are service providers in various capacities – some of these members kindly provided insights into their work and shared the benefits of being a person with lived experience who is providing a mental healthcare service.

### Virtual support group facilitator: Sandra Ferreira (South Africa)

‘I facilitate online mental health support groups during the COVID-19 pandemic. The virtual platform has provided participants from across the world the opportunity to voice their experiences during the pandemic and discuss the impact on their mental health.

One of the most interesting observations that I have drawn from this experience was that many of the participants were actively involved in advocacy and awareness work in their respective countries. This is not uncommon as our struggles often lead to the need to better the road for those that may follow, aligned with a need to be relevant, to be valued and to make a difference. Essentially, giving us a purpose.

In its purest form, this virtual support group has been a space to “unmask”, to breathe easy, and to be free – not only during current struggles of dealing with the pandemic and the restrictions it has imposed on our daily lives but also to just be yourself without judgment.

The greatest benefit from providing a support service is the validation that through helping others, we are helping ourselves become better human beings, more knowledgeable advocates, and most importantly, more empathetic supporters of humankind.’

### Founder of an online peer support platform (CARA Unmask): Bernard Ang (Singapore)

‘Reflecting back on my journey with depression, I wish I could have opened up about my mental health issues to someone who listened and understood. My experiences led to me founding CARA Unmask (caraunmask.com), an online peer support platform that encourages people to reach out earlier rather than later, to have a chat about their mental health. We recognize the direct benefits that come from wanting to share valuable lived experiences, which creates purpose, cultivates a sense of gratitude, facilitates personal growth and simply paying it forward. Peer support is evidence-based and proven to work effectively, side-by-side with clinical support for a sustainable long-term recovery outcome.’

### Peer support specialist: Syd Gravel (Canada)

‘It was worrisome at first – that first peer meeting. Knowing how complex and confusing things had been for me being mentally injured by a traumatic incident at work. Now, the psychologist was asking me if I would be willing to meet others who were also injured in similar situations. The goal was to see how we could help each other by sharing our experiences so that we would realize that we were not alone. I was not even sure I knew how to help myself let alone someone else.

I wondered how this conversation could even start – how was this meeting going to help me? How could sharing my situation help someone else?

That was 32 years ago and since that evening of our first peer support meeting, I have never looked back, as we created a bond that never waned. We are in touch with each other, to this day. I am now a full-time consultant on trauma management and peer support development for First Responder agencies and author to several books on the subject. Amazing isn't it, how when life throw us lemons, we can learn to make lemonade.’

### Social worker: Thandiwe Mkandawire (Zimbabwe)

‘During my training as a clinical social worker, my therapist once said to me “We all become therapists because we all have psychological pain we are trying to heal, to find our true selves and in doing this work, not only do we help others, we also help ourselves”. The healing of the mind, as is any form of healing, is painful and difficult as it takes honesty, vulnerability, courage and bravery to face your emotions and engage in the necessary war of fighting the rhetoric in your mind.

Working in the field of mental health and listening to service users and carers share their stories and their truths in support groups, at awareness campaigns or clinic days at the hospitals, fighting through self-stigma and societal stigma and discrimination, allowed me to realize that experiences are as unique as the person, a person's truth cannot be classified as greater or lesser than the next person's. My experience is my truth and it needs to be shared’.

### Advanced lived experience practitioner: Mark Sanderson (UK)

‘I have worked in mental health services since 2016. I started as a volunteer on an inpatient ward, where I had been a previous inpatient. After 6 months of volunteering, I was employed into a paid peer support role. Within my first year I won a runner up prize for innovation, which boosted my confidence. I continued to grow in the role and was constantly given opportunities to contribute to service development and presented at various conferences and training sessions. I am currently pursuing a Master's degree in Mental Health Recovery and Social Inclusion.

I continued to advance in my career and obtained a non-peer position, which involved supporting the discharge process of service users from the ward into the community. Alongside this role I worked with the senior management team to develop a senior peer support position within the inpatient setting and simultaneously was working with another manager to develop a senior peer support position for the community.

In around four and a half years I have worked my way from a volunteer to an Advanced Lived Experience Practitioner and have found my studies to play an essential role in my development. Moving forward I aim to continue developing in lived experience roles within the NHS.’

### Psychiatrist: Raluca Mirela (Romania)

‘Working in child and adolescent psychiatry as a person with lived experience and as a former victim of psychological and physical child abuse was often more of an emotional curse than a benefit, because I deeply empathise with the children for whom I felt responsible. My frustrations resulted from working with families that were not used to a bio-psychosocial approach – and they often asked me why I am talking so much about their child, because they just wanted the medical certificate (in order to apply for disability aid). They were not familiar with taking children to other specialists like neurologists, paediatricians, or to psychotherapy, and mostly refused to do so by justifying that they do not have money or time (even if they receive a paid medical leave to take their children to the doctor). Despite the emotional burden, working with compliant families and seeing the improvement in their child's health (mentally and emotionally) gives me enormous joy and a motivational boost.’

## Discussion

Global recognition of the importance of the role of persons with lived experience in mental healthcare has gained momentum, with academics, clinicians, researchers and mental health organisations placing emphasis on improving the status quo through peer support systems and improved service delivery.

Caution, however, must be exercised in our approach and we must take into account the implications of the diverse experiences of individuals in their mental health recovery processes and recognise that ‘many people with lived experience lack the confidence or ability to articulate their views, particularly if they contradict the status quo and especially when speaking to people who hold similar roles to those who have taken choices away from us’.^6^

Nevertheless, there is no denying that there is potential for leadership development of persons with lived experience in mainstream industries. Mental healthcare organisations ought to take the lead in driving initiatives by wisely employing the insights of persons with lived experience. Strategies for peer support-based service delivery is encouraged because having the support of a peer who has been exposed to similar experiences has a way of allowing for meaningful conversation in a safe environment. The conventional hierarchy of clinician and client or patient does not apply in peer support systems, as the focus is placed on peer relationships and the development of trust among peers. The challenge, however, lies in ensuring that the discipline of lived experience in mental healthcare does not become too codified or regulated, as it will lose its real purpose and lose what is the most important reason for someone's existence.^7^

It is likely that people who enter the field of mental healthcare do so because of their desire to help others and often also because of their personal experience of mental health conditions. In principle, having lived experience and having knowledge of service delivery and its corresponding processes provides them with an ‘advantage’ of being in a better position to step into another's shoes and be truly empathetic to their clients’ or patients’ needs and vulnerabilities. In most cases people choose to become therapists to make a difference in someone else's life, to be appreciated for making a difference in society and to help others who have struggled with similar painful experiences as they did.^8^

Given the above, by empowering persons with lived experience by means of education and by developing and maintaining leadership roles there will undoubtedly be a positive move towards improvement in mental health recovery. Therefore, stakeholders are encouraged to further explore the value of peer support as well as the personal experiences of clinicians for improved mental health recovery and improved quality of service delivery. Lastly, all stakeholders must ensure that they take into consideration the inputs and recommendations of persons with meaningful and authentic lived experience and implement them in their strategies and policies.

## About the authors

**Charlene Sunkel** is founder and chief executive officer (CEO) of the Global Mental Health Peer Network, Roodepoort, South Africa. **Claudia Sartor** is deputy CEO of the Global Mental Health Peer Network, Roodepoort, South Africa.
